# Morphometric parameters and food preference in relation to sex and reference hematological values for *Upupa epops* from Pakistan

**DOI:** 10.5455/javar.2022.i595

**Published:** 2022-06-27

**Authors:** Rida Tahir, Warda Zafar, Muhammad Waseem Aslam, Ahmad Waheed, Ali Umar, Sana Fatima, Tariq Javed, Tabish Liaqat, Allah Ditta, Muhammad Ashfaq, Muhammad Zaman, Ali Nawaz, Tehmina Khan, Muhammad Wajid, Muhammad Saleem Khan

**Affiliations:** 1 Department of Zoology, Faculty of Life Sciences, University of Okara, Okara, Pakistan; 2 Department of Zoology, Wildlife and Fisheries, University of Agriculture, Faisalabad, Pakistan; 3 Department of Fisheries and Aquaculure, Faculty of Life Sciences, University of Okara, Okara, Pakistan

**Keywords:** Common hoopoe, gut contents, hematology, morphometry, Pakistan

## Abstract

**Objective:**

The study was conducted to investigate the gut content and record morphometric and hematological parameters in the common hoopoe (*Upupa epops*).

**Materials and Methods:**

Twenty samples of healthy birds (10 from each sex) were collected from different locations in Okara District, Punjab, Pakistan, from September 2020 to March 2021. Birds were captured live for blood samples and morphometric and gut analyses.

**Results:**

It was revealed that the concentrations of different hematological parameters were as follows: hemoglobin, 20.03g/dl; red blood cells, 3.28 × 106/µl; white blood cells, 326.67 × 103/µl; hematocrit, 56.47%; MCV, 173.33 FL; MCH, 57.4 pg; MCHC, 57.4 pg; PLT, 8.33/µl; and RDW, 8.33/µl. The percentages of neutrophils, lymphocytes, monocytes, and eosinophils were 84.67%, 11.67%, 2.00%, and 1.67%, respectively. The gut content of the common hoopoe mostly consisted of Coleoptera and Acrididae larvae. However, Lepidoptera, Gryllotalpidae, and sand were also recorded, along with seeds of *Salvadora persica*.

**Conclusions:**

There were no significant differences between male and female *U. epops* in feeding content, total weight of the gut, or weight of the empty gut. Regarding the morphometric parameters, there was a significant difference in both sexes’ wingspan, body length, and body weight. Males were significantly heavier than females.

## Introduction

The common hoopoe (*Upupa epops*) is known by its Arabic name, “*Hudhud*,” in Pakistan. It is a remarkable and unique old-world bird that belongs to the family Upupidae. It has its foraging style with special external features [1]. The distribution ranges from Europe and North and sub-Saharan Africa (including Madagascar) to Asia. They mainly breed in Europe, Africa, Malaysia, the Middle East, China, and Indonesia [2]. Most of the African and Southeast Asian populations of common hoopoe remain in their native areas in the winter and do not migrate [3].

They also migrate to the tropical region from North Asia and Europe during the winter seasons [4]. This bird is common in Pakistan and reaches Pakistan by flying over the Karakoram and Sulaiman ranges and the Hindu Kush beside the Indus River [5]. It is also a summer-breed visitor in the northern Himalayas and Indus plains [2]. The best living places are wooded steppes, savannas, grasslands, and forest glades. Deserts and natural forests are avoided [2,6].

*Upupa epops* is a small to medium-sized, slender-shaped bird with a 29–31 cm body length and a 64–77 gm weight in males and 57–69 gm in females [7]. The head is mainly rufous orange to orange-brown or salmon pink in color, with sticking black and white wings of an average length of 13.6–15.3 cm [8]. During flight, the upper surface shows alternative white and black bars. *Upupa* epops has a long, thin, downcurved black bill of 5–6.3 cm and a squared tipped, black and white striped tail of 9.8-10.9 cm [7]. Like the erectile crest crown, the high black tip fan consists of 28 feathers (long, narrow, and orange) on the common hoopoe’s head [9]. They are usually held flat, curved downwards in the narrow tail in the rest position, and in excited or alarmed conditions, the crest is erect and fan-shaped [2,7].

Avian hematology started early in the 1960s. In veterinary practice, hematology is vital, and changes in avian blood’s morphology and composition help detect and diagnose health issues [10]. The blood parameters change in response to health status and migration. For example, hemoglobin concentration increases significantly over the migratory period [11]. In many species, hemoglobin concentration starts to grow from the time of fledgling until adulthood [12]. Some species, along with heterophils, respond to stress with lymphocytosis (increased lymphocytes). In the case of any chronic disease, the number of monocytes increases, while in allergic or parasitic conditions, the number of eosinophils increases [13]. Therefore, building standard reference values is necessary for each avian species.

The common hoopoe is mostly insectivorous; it feeds on small worms (annelids), larvae of ant-lions (*Myrmeleonidae*), Elaleid beetles Agrotis larvae, Hemiptera bugs, etc. In some studies, small reptiles, frogs, and plant matter were also recorded in the food [2,6]. Hoopoes that inhabit farmland mainly feed on mole crickets (Gryllotalpidae) and Lepidoptera larvae [14], whereas hoopoes in pine plantations mainly feed on pupae of the pine moth (*Thaumetopoea pityocampa*) [6,15]. During the winter–autumn season, they feed on ants (*Componotus compressces*). The primarily young feed on soil invertebrates [2,16]. The study was conducted to investigate the gut content and record morphometric and hematological parameters in common hoopoe (*U. epops*).

## Materials and Methods

### Ethical approval

All procedures carried out on the animals in this study followed the rules set by the University of Okara’s Ethical Committee (approval number: UO/DOZ/2020/misc.).

### Study area and sampling

Samples of the common hoopoe were collected from the grassy wooded steppes area of Renala Khurd (30.88°N, 73.60°E), Pipli Pahar (30.68°N, 73.43°E), and alongside the Ravi river in Okara District. These places were visited in the morning and evening from December 2020 to March 2021, twice a day for sampling. Twenty samples of the common hoopoe (10 from each gender) were captured with the help of local hunters using a net. After capture, we anesthetized the birds by using a combination of ketamine HCL (10 mg/kg) and diazepam (0.2 mg/kg) [17].

### Blood sample analysis, gut content, and morphometry

Analyses of hematological and morphometric characteristics and gut content of the common hoopoe were carried out according to the methodology described by Aslam et al. [18].

### Statistical analysis

Data were analyzed through mean, standard deviation (SD), standard error (SE), and range using GraphPad Prism 9.0 software. The significant difference was tested through an unpaired *t*-test at a 0.05 confidence level.

## Results and Discussion

### Hematology

The hematological values are used to indicate the health state of birds, as well as mammals. These are used for diagnosing and monitoring diseases, evaluation of disease therapy, or disease prognosis. These can also be used as physiological reference values for specific indicators for different bird species. Different physiological factors can affect the hematology of healthy birds [19,20]. The present study provides physiological reference values for normal values of the adult birds of this species (Table 1). No previous comparable records are present for the species in the present analysis.

**Table 1.** Hematological parameters of the common hoopoe collected from Okara District, Punjab, Pakistan.

Variable	SE	Mean ± SD
HGB (gm/dl)	2.16	20.03±3.73
WBC (×10^3^/µl)	2.88	326.33±3.33
RBC (×10^6^/ µl)	0.22	3.28±0.37
HCT (%)	0.41	56.47±0.70
MCV (FL)	0.72	173.33±1.25
MCH (pg)	0.71	58.97±1.23
MCHC (gm/dl)	1.66	34.33±2.87
PLT (×10^3^/ µl)	1.19	8.33±2.05
RDW	1.28	75.00±2.21
Neutrophils	2.60	84.67±4.50
Lymphocytes	3.31	11.67±5.73
Monocytes	0.47	2.00±0.82
Eosinophils	0.27	1.67±0.47

SE = Standard error; SD = Standard deviation.

**Figure 1.**
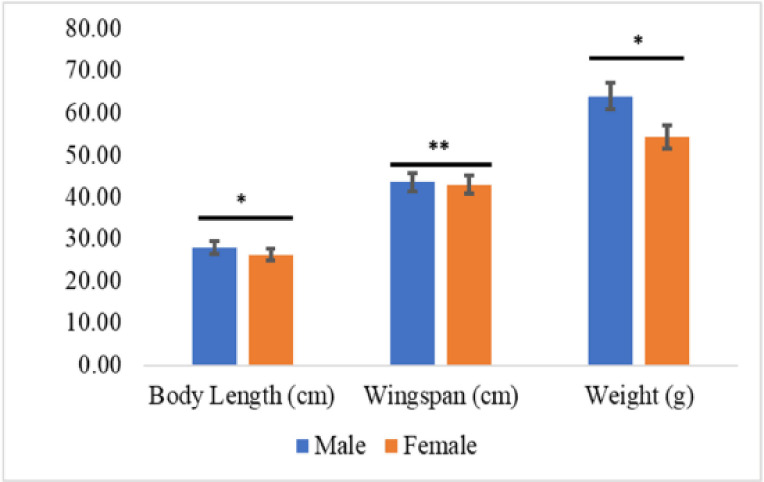
Comparison of body length, wingspan, and weight of both sexes of *U. epops* (*p < 0 .05; **p < 0.01)

**Table 2.** Comparison of morphometric characteristics between male and female *Upupa epops* collected from Okara District, Punjab, Pakistan.

Characters	Sex(*n* = 10 each)	SE	Mean	Range	*p*-value
Body weight (gm)	Male	2.07	64.03	57.70–68.00	0.01*
	Female	1.04	54.35	52.60–57.70	
Body length (cm)	Male	0.12	27.88	27.50–28.10	0.01*
	Female	0.21	26.23	26.40–27.50	
Tail length (cm)	Male	0.14	11.18	10.80–11.50	0.175^NS^
	Female	0.11	10.85	10.50–11.10	
Wingspan (cm)	Male	0.06	43.70	43.60–43.90	<0.01**
	Female	0.03	43.05	43.00–43.10	
Wing length (cm)	Male	0.13	18.85	18.40–19.10	0.142^NS^
	Female	0.08	18.55	18.40–18.70	
Longest primary feather (cm)	Male	0.12	13.53	13.20–13.90	0.518^NS^
	Female	0.02	13.43	13.40–13.50	
Tarsus (cm)	Male	0.12	2.40	2.00–2.60	0.115^NS^
	Female	0.05	2.13	2.00–2.30	
Central toe length (cm)	Male	0.03	2.15	2.10–2.20	0.228^NS^
	Female	0.04	2.08	2.00–2.20	
Head length without bill (cm)	Male	0.08	3.25	3.10–3.50	0.085^NS^
	Female	0.14	2.88	2.60–3.20	
Head length with bill (cm)	Male	0.01	8.75	8.60–8.90	0.055^NS^
	Female	0.13	8.35	8.10–8.60	
Bill length (cm)	Male	0.04	5.50	5.40–5.60	0.620^NS^
	Female	0.02	5.48	5.40–5.50	
Chest circumference (cm)	Male	0.17	13.78	13.40–14.20	0.007^NS^
	Female	0.09	12.90	12.60–13.10	

* *p* <0 .05;

** *p* < 0.01; NS = Nonsignificant difference (*p*-value > 0.05).

**Table 3.** Weight of gut variables in male and female common hoopoes.

Characters	Gender	*N*	Mean	SD	SE	*t*-value	*p-*value
Total weight of gut (gm)	Male	10	2.30	0.62	0.36	0.01	0.9512^NS^
	Female	10	2.33	0.37	0.22		
Weight of food material (gm)	Male	10	1.06	0.49	0.28	0.02	0.9052 ^NS^
	Female	10	1.01	0.33	0.11		
Weight of empty gut (gm)	Male	10	1.24	0.13	0.07	0.74	0.4163^NS^
	Female	10	1.32	0.04	0.33		

NS = Nonsignificant difference (*p*-value > 0.05).

**Table 4.** Gut content of male and female common hoopoes.

Type of Food	Weight of different gut contents (%)		*p*-value
	Male	Female	
Lepidoptera	0.00	10.33	0.3090^NS^
Gryllotalpidae	0.00	4.00	0.3739^NS^
*S. persica*	15.33	18.33	>0.9999^NS^
Acrididae	0.00	2.67	0.3739^NS^
Coleoptera	61.67	24.00	0.0866^NS^
Sand	0.00	5.33	0.3739^NS^
Digested material	23.00	35.33	0.4012^NS^

NS = Nonsignificant difference (*p*-value > 0.05).

### Morphometry

Significant differences were observed in wingspan, body weight, and body length (Fig. 1), while all other morphometric parameters were nonsignificant (Table 2).

Morphological analysis helps understand the evolutionary processes [21,22]. The present study was similar to David [23] and Roberts [2] in the morphometric measurement of body weight, body length, wingspan, and length of the longest primary feather of male and female common hoopoes. In the case of bill length, similar values to our study were also reported by van Wijk et al. [7], Elshaer [24], and Roberts [2]. At the same time, all other remaining morphometric parameters were reported for the first time in the present study.

### Food preferences

There were nonsignificant differences between total weight of the gut, weight of the food content, and weight of the empty gut for both sexes (Table 3). The gut analysis shows that the common hoopoe feeds mainly on Coleoptera and Acrididae larvae. However, Lepidoptera, Gryllotalpidae, and sand were also found. The seed of *Salvadora persica* was found in the gut. The difference in feeding content between both sexes was nonsignificant (Table 4).

The study of feed preference is important from ecological and conservation perspectives [25]. The gut content of *U. epops* consisted of larvae of Lepidoptera, Gryllotalpidae and Acrididae, Coleoptera, and sand, which were also reported by Kristin [6] and Roberts [2]. We also found some plant matter, i.e., seeds of *S. persica*, in the gut of *U. epops*, which Fournier and Arlettaz [14] also reported. However, our outcomes differed from Roberts [2] in the case of small reptiles and frogs, as we did not find any of these in the gut content of *U. epops.* Namma and Rao [26], Myo et al. [27], and Tomás et al. [28] reported that *U. epops* preferred to eat insects. This difference might be due to the difference in habitat or food availability.

## Conclusion

The morphometries of both sexes of the common hoopoe (except for body weight, body length, and wingspan), gut weight, and gut content were similar. Males were larger and heavier as compared to females. The gut analysis shows that the common hoopoe feeds on Coleoptera, Acrididae larva, Lepidoptera, Gryllotalpidae, sand, and seeds of different plants, such as *S. persica*. This study discusses all blood parameters of the common hoopoe for the first time.

## List of abbreviations

HGB, Hemoglobin; WBC, White blood cells; RBC, Red blood cells; HCT, Hematocrit; MCV, Mean corpuscular volume; MCH, Mean corpuscular hemoglobin; MCHC, Mean corpuscular hematocrit; PLT, Platelets; RDW, Red cell distribution width.
